# *RTA1* Is Involved in Resistance to 7-Aminocholesterol and Secretion of Fungal Proteins in *Cryptococcus neoformans*

**DOI:** 10.3390/pathogens11111239

**Published:** 2022-10-26

**Authors:** Emily S. Smith-Peavler, Ronakkumar Patel, Adejumoke Mary Onumajuru, Bethany G. Bowring, Joyce L. Miller, Jean Michel Brunel, Julianne T. Djordjevic, Moses M. Prabu, Erin E. McClelland

**Affiliations:** 1 Department of Biology, Middle Tennessee State University, Murfreesboro, TN 37132, USA; 2 Otsuka Pharmaceutical, 508 Carnegie Center Dr., Princeton, NJ 08540, USA; 3 Centre for Infectious Diseases and Microbiology, The Westmead Institute for Medical Research, Sydney, NSW 2145, Australia; 4 MTSU Interdisciplinary Microanalysis and Imaging Center, Middle Tennessee State University, Murfreesboro, TN 37132, USA; 5 UMR_MD1 “Membranes et Cibles Thérapeutiques”, Faculté de Pharmacie, Aix Marseille Universite, INSERM, SSA, MCT, 13385 Marseille, France; 6 Sydney Institute for Infectious Diseases, University of Sydney, Sydney, NSW 2145, Australia; 7 M&P Associates, Inc., Murfreesboro, TN 37129, USA; 8 Biomedical Sciences Division, Marian University College of Osteopathic Medicine, Indianapolis, IN 46234, USA

**Keywords:** *Cryptococcus neoformans*, *RTA1*, secretion, extracellular vesicles

## Abstract

*Cryptococcus neoformans* (*Cn*) is a pathogenic yeast that is the leading cause of fungal meningitis in immunocompromised patients. Various *Cn* virulence factors, such as the enzyme laccase and its product melanin, phospholipase, and capsular polysaccharide have been identified. During a screen of knockout mutants, the gene resistance to aminocholesterol 1 (*RTA1*) was identified, the function of which is currently unknown in *Cn*. Rta1 homologs in *S. cerevisiae* belong to a lipid-translocating exporter family of fungal proteins with transmembrane regions and confer resistance to the antimicrobial agent 7-aminocholesterol when overexpressed. To determine the role of *RTA1* in *Cn*, the knock-out (*rta1Δ*) and reconstituted (*rta1Δ+RTA1*) strains were created and phenotypically tested. *RTA1* was involved in resistance to 7-aminocholesterol, and also in exocyst complex component 3 (Sec6)-mediated secretion of urease, laccase, and the major capsule component, glucuronoxylomannan (GXM), which coincided with significantly smaller capsules in the *rta1Δ* and *rta1Δ+RTA1* strains compared to the wild-type H99 strain. Furthermore, *RTA1* expression was reduced in a secretory 14 mutant (*sec14Δ*) and increased in an RNAi Sec6 mutant. Transmission electron microscopy demonstrated vesicle accumulation inside the *rta1Δ* strain, predominantly near the cell membrane. Given that Rta1 is likely to be a transmembrane protein located at the plasma membrane, these data suggest that Rta1 may be involved in both secretion of various fungal virulence factors and resistance to 7-aminocholesterol in *Cn*.

## 1. Introduction

*Cryptococcus neoformans* (*Cn*) is an encapsulated fungal pathogen that predominantly affects immunocompromised individuals, such as individuals infected with HIV or organ transplant recipients who are taking immunosuppressants [1]. In host organisms, a variety of health outcomes have been observed. In most immunocompetent patients, once basidiospores or desiccated yeast cells of the pathogen have been inhaled, the immune system is able to resolve or control the infection before any detrimental effects are observed. In immunocompromised patients, however, dissemination from the lungs to the central nervous system (CNS) often results in meningitis [1]. Cryptococcal infections lead to more than 220,000 new cases and 181,000 deaths every year [2]. This number has been lowered significantly in the last few years due to the use of antiretroviral drugs against HIV and early administration of antifungals for patients who become immunocompromised.

A number of virulence factors in *Cn* have been characterized, including melanin produced by the fungal enzyme laccase, phospholipase B, urease, and the polysaccharide capsule, as well as the shedding of immunomodulatory capsular polysaccharide [3]. Genes implicated in the production and secretion of these virulence factors are of interest for their potential as therapeutic targets and knowledge of how they affect pathogenesis.

The gene Resistance to aminocholesterol 1 (*RTA1*, CNAG_03091) was identified in *Cn* during a screen of knockout mutants that did not make melanin (Figure S1). However, its function and role in melanization and *Cn* virulence is currently unknown. The *RTA1* homolog in *Saccharomyces cerevisiae* belongs to a lipid-translocating exporter family with transmembrane regions (transporter classification [TC] 9.A.26.1.1) and its overexpression confers resistance to the antimicrobial sterol 7-aminocholesterol [4].

Aminosterols, including the drug 7-aminocholesterol, are of interest in antifungal drug development for their potential activity against reference yeast strains, including *Candida albicans* and *Cn* [5]. 7-aminocholesterol inhibits yeast cell proliferation via a mechanism that is not fully understood. However, it is thought to be a morpholine-like compound, which suggests it may be involved in inhibition of the ergosterol biosynthesis pathway [6]. In this pathway, 7-aminocholesterol is thought to inhibit the function of the Erg24 and Erg2 enzymes, encoding a C-14 sterol reductase [7] and a C-8 sterol isomerase [8], respectively, leading to the accumulation of ergosterol intermediates (including fecosterol) [6]. Since ergosterol is the major sterol in the Basidiomycota cell membrane [9], disruption of the ergosterol biosynthesis pathway may cause the cell membrane of *Cn* to become more permeable, which may lead to cell death [6].

In *Cn*, virulence factors are exported from the cell via one or more secretory pathways. The exocyst complex component 3 (Sec6) pathway is involved in fusion of exocytic vesicles with the plasma membrane and has been implicated in laccase as well as urease and glucuronoxylomannan (GXM) secretion [10,11,12]. The Sav1/Sec4 pathway is involved in vesicle-mediated exocytic secretion and has been implicated in GXM secretion [13,14]. The secretory 14 (Sec14) pathway is involved in specific endosome and non-endosome-dependent secretion pathways originating in the Golgi and has been implicated in phospholipase B1 secretion [15,16] and acid phosphatase secretion [17]. While research on secretion in *S. cerevisiae* [18] has helped elucidate secretory mechanisms in *Cn* [12,13,16,19], the precise mechanisms are not fully understood, especially the mechanism by which extracellular vesicles are transported out of the cell [12].

Given the putative role of *RTA1* in melanization in *Cn* and its role in conveying resistance to 7-aminocholesterol from studies conducted in *S. cerevisiae*, *Cn RTA1* deletion and *RTA1*-reconstituted strains were created and characterized. Specifically, whether *RTA1* was involved in resistance to 7-aminocholesterol and secretion of virulence factors, including laccase, urease, and GXM, was investigated using phenotypic determination and transmission electron microscopy.

## 2. Materials and Methods

### 2.1. Strain Construction

The wildtype strain utilized in this study was *Cryptococcus neoformans sensu stricto* H99S (a kind gift from John Perfect, Duke University). *RTA1* knockout strains (KO22) and reconstituted strains (KI66) were created using overlap polymerase chain reaction (PCR) and plasmid delivery, respectively. Cells were cultured in yeast peptone dextrose (YPD, Thermo Fisher, Waltham, MA, USA catalog #DF0428-17-5) medium unless otherwise noted. Both 7-aminocholesterol and the fluorescently labeled dansyl 7-aminocholesterol used in this study were synthesized by J.M. Brunel. 7-aminocholesterol was stored in powder form at room temperature out of direct light until resuspended in dimethyl sulfoxide for all experiments.

Knockout constructs were created by cloning the nourseothricin (*NAT*) resistance marker and the flanking regions of *RTA1* into the TOPO vector (Thermo Fisher Scientific, Waltham, MA, USA, catalog #K465001). Flanking regions were amplified from the H99 wildtype strain and the *NAT* cassette was amplified from the pPZP-NATcc plasmid (a kind gift of Dr. Joe Heitman, Table S1). The left flanking regions and *NAT* cassette (left + *NAT*) were cloned into the pUC21 plasmid (a kind gift of Dr. Helene Barbour). The right flanking region and left + *NAT* were amplified and joined using overlap PCR. This construct was then cloned into the TOPO vector and the sequence confirmed through sequencing (Figure S2a). Plasmid DNA was harvested from transformed *Escherichia coli* (*E. coli*) cells and the construct was amplified. Reconstitution constructs were created by cloning the neomycin (*NEO*) resistance marker, *RTA1*, and flanking regions into the pAllet plasmid (a kind gift from Dr. J. Brian Robertson, Figure S2b), followed by transformation into *E. coli* cells. *RTA1* and its flanking regions were amplified from the H99 wildtype strain and the *NEO* cassette was amplified from the pJAF plasmid (a kind gift of Dr. James Fraser, Table S1). Transformation was confirmed through PCR, restriction digest, and positive growth on media containing NAT (Werner Bioagents, Jena, Germany, catalog #5.001.000) or NEO (Sigma Aldrich, St. Louis, MO, USA, catalog #D1720). Finally, the knockout construct was introduced into the H99 wild type strain, resulting in the *rta1Δ* strain, and the reconstituted plasmid construct was introduced into the *Cn rta1Δ* strain, resulting in the *rta1Δ+RTA1* strain through biolistic transformation and homologous recombination.

Cells were plated onto YPD plates containing NAT (200 µg/mL) or NEO (200 µg/mL) and incubated for 48 h at 37 °C. Colonies were patched onto a secondary YPD+Nat or YPD+Neo plate (200 µg/mL) and incubated for 48 h at 37 °C. Colonies were serially passaged five times on non-selective YPD to ensure successful integration of the construct into the genome. Colonies were patched on one final selection plate and then colonies that grew were frozen down and stored at −80 °C. Positive knock out and reconstituted strains were confirmed through PCR and Southern blot (Figure S3) using the DIG DNA labeling and detection kit (Roche, Basel, Switzerland, catalog #11093657910).

### 2.2. Resistance to 7-Aminocholesterol

Resistance to 7-aminocholesterol was measured as previously described in [4]. Strains were grown in YPD to log-phase, washed three times in phosphate-buffered saline (PBS), and 1 × 10^6^ cells/mL were diluted in PBS. Cells were serially diluted to 10^−1^, 10^−2^, and 10^−3^ cells/mL, and 10 µL of each cell concentration was spotted onto YPD + 7-aminocholesterol plates (0.5, 1, and 2 µg/mL). The plates were incubated at 37 °C for 3 days and the presence/absence of growth was examined.

### 2.3. Capsule Induction

Capsule thickness in vitro was determined as previously described [20,21]. Briefly, 1 × 10^5^ cells/mL of H99, *rta1Δ*, and *rta1Δ+RTA1* were added to Dulbecco’s modified Eagle medium (DMEM, Invitrogen, Waltham, MA, USA, catalog #10566-016) and incubated at 37 °C + 10% CO_2_ for 18 h. Cells were collected, suspended in 10 μL PBS and added to a microscope slide with India Ink (Fisher Scientific, Waltham, MA, USA, catalog #14-910-56). Cells were imaged on an Eclipse TS100 Nikon microscope (Tokyo, Japan) with a 100× objective. For each strain, pictures of 50–70 cells were captured. The diameter of the cell body and capsule were measured using Zeiss AxioVision software, v4.9.1. Capsule thickness was calculated by subtracting the cell body diameter from the diameter of the entire cell + capsule and dividing by 2.

### 2.4. Capsule Shedding

To determine if the strains differed in their ability to release capsular glucuronoxylomannan (GXM) into the medium, capsules were induced in DMEM at 37 °C + 10% CO_2_ for 18 h (as for measuring capsule size). The next day, the DMEM supernatant was collected, the concentration of GXM in the media was measured by GXM capture enzyme-linked immunosorbent assay (ELISA) and the absorbance was measured at 405 nm, as previously described [22].

### 2.5. Melanin Production

Melanin production was determined as in [23]. The H99 and *rta1Δ* strains were grown in YPD to log-phase, washed three times in PBS, and 1 × 10^6^ cells/mL were diluted in PBS. Cells were serially diluted to 10^−1^, 10^−2^, 10^−3^, 10^−4^ and 10 µL of each cell concentration was spotted on 1 mM L-DOPA (Sigma Aldrich, St. Louis, MO, USA, catalog #D9628) plates. The plates were incubated at 30 °C for 3–5 days and then checked for melanization.

### 2.6. Urease Production

The urease activity of the H99, *rta1Δ*, and *rta1Δ+RTA1* strains was determined as previously described [24]. Briefly, strains were grown in YPD to log-phase, washed three times in PBS, 5 × 10^7^ cells were suspended 1:1 with PBS and 2× Roberts Urea broth and incubated at 37 °C for 4 h. After 4 h, the strains were centrifuged to pellet the cells and the supernatant was measured by spectrophotometry at 560 nm on an M5 spectrophotometer (Molecular Devices, San Jose, CA, USA). Urease production was indicated by pink color in the supernatant.

Urease secretion was also determined in the *sec14Δ* strains. Cells were grown in YPD overnight, washed twice with distilled water, 1 × 10^7^ cells/mL were suspended 1:1 with distilled water and 2× Roberts Urea broth, and incubated at 37 °C, shaking, for 3 h 30 min. The strains were then centrifuged to pellet the cells and the absorbance of the supernatant was measured at 560 nm.

### 2.7. Laccase Production

Laccase secretion was measured as previously described [25]. Strains were grown in YPD to log-phase, washed twice in PBS, once in asparagine media with glucose, and then 2 × 10^8^ cells were transferred to 10 mL asparagine media with glucose. Cells were grown overnight at 37 °C and then washed twice with PBS and once with asparagine media without glucose.

Cells were grown overnight at 37 °C, washed twice with Britton-Robinson buffer [pH = 5.0 (BRB)] [25], counted using a hemocytometer, and 1 × 10^6^ cells were added to 900 µL BRB buffer with 100 µL 10 mM 2,2′-azino-bis (3-ethylbenzothiazoline-6-sulfonic acid) diammonium salt (Sigma Aldrich, St. Louis, MO, USA catalog #A1888) in triplicate. The cells were incubated at 25 °C, with shaking for 30 min and the absorbance was measured at 415 nm.

### 2.8. Phospholipase B Secretion

Extracellular phospholipase secretion was measured as previously described [26]. The H99 and *rta1Δ* strains were grown in YPD to log-phase, washed three times in PBS, and then 50 µL of a 1 × 10^3^ cell/mL dilution were plated onto two Malt Egg Yolk Agar plates per strain. The plates were incubated at 30 °C for 10 days. After 10 days the plates were photographed using an Alfa Imager with a color filter. The phospholipase index was calculated for 15 colonies for each strain. The diameter of the colony plus the diameter of the precipitation zone was measured using Adobe Photoshop, San Jose, CA, USA. The phospholipase index is the ratio of the diameter of the colony to the diameter of the colony plus the precipitation zone.

### 2.9. Intracellular 7-Aminocholesterol Visualization

To determine if trapped 7-aminocholesterol could be visualized in the *rta1Δ* strain, the strains were grown in the presence of fluorescent dansyl 7-aminocholesterol and then stained with Congo red (Sigma Aldrich, St. Louis, MO, USA, catalog #C6277) to visualize the cell wall. The H99, *rta1Δ*, and the *rta1Δ+RTA1* strains were inoculated in YPD broth and grown to log phase at 37 °C. The cells were washed three times with PBS and counted. A total of 1 × 10^6^ cells were diluted in YPD containing 1.0 µg/mL fluorescent dansyl 7-aminocholesterol and shaken at 150 RPM and 37 °C for 16 h. Cells were then pelleted and washed with PBS. Cells were treated for 10 min with a 0.1% solution of Congo red and observed using fluorescence microscopy with a Nikon Ti-Eclipse inverted wide field microscope and an oil-emersion 63× objective (Nikon, Tokyo, Japan).

### 2.10. Homology Modeling

Protein modeling was conducted to interpret the fluorescence microscopy results in the context of structure function because three-dimensional information for Rta1 and its homologs were unavailable. *Cn* Rta1 is hypothesized to have seven transmembrane helices (Figure S4). Preliminary protein modeling to determine secondary structure was completed using Quick2D software [27] and PsiPred 2D modeling [28] to identify theoretical alpha helix, beta sheet, and transmembrane units within the Rta1 protein. In addition, a potential tertiary structure of Rta1 using NetWheels was generated to model the orientation and polarity of the predicted alpha helices [29]. Those were compared to proteins with similar secondary structures for which a three-dimensional structure was known. Additional secondary structural predictions were performed using the online interface PredictProtein [30] and the predictions found to be common were used for this analysis.

While amino acid sequence similarity may indicate functional similarity, sequence similarity is not required for proteins to be structurally or functionally similar [31]. The Rta1 amino acid sequence for *S. cerevisiae* is different from that in *Cn*, but both confer resistance to 7 aminocholesterol [4]. In addition, it has been shown in GTPase protein domains that, while there is usually sequence similarity in binding sites, the rest of the protein may have low sequence similarity and still have functional similarity [32,33]. There were no proteins identified with significant sequence similarity to Rta1. However, other proteins from the protein data bank (PDB) that also had seven transmembrane helices, similar predicted gene ontologies, such as pores or channels [34], and were of similar size to Rta1 were short listed. This analysis identified six proteins (Table 1).

Using PsiPred, those PDB structures were probed for conserved helical topology and the list of structural hits were further narrowed to four (4OR2, 3RKO, 5G28, and 3AG3). A structural model for *Cn* Rta1 was generated by threading the Rta1 sequence over the crystal structure using conserved patterns within the helical domains. The backbone atoms along with the positions of the beta carbons were used in positioning the Rta1 side chains. The molecular modeling program UCSF Chimera [35] was used in model building. Stretches of Rta1 sequence that did not share equivalents in the PDB structures were not modeled. Local minimization for stretches of 10 amino acids was performed to relax the side chains. Steric clashes were removed and local minimization was done iteratively until the modeled side chains did not make bad contacts. To further pare down the potential structural templates to be used for Rta1, these proteins were also analyzed for orientation of the helices within the membrane. The chloride-pumping rhodopsin (CIR) protein (PDB: 5G28) has a similar secondary structure to Rta1, seven transmembrane regions in alpha helical formations [36], and function, which is analogous to the hypothesized function of Rta1 transporting 7-aminocholesterol out of the cell.

### 2.11. Phylogenetic Analysis

A phylogenetic maximum-likelihood tree with 1000 bootstraps of fungal Rta1 amino acid sequences was created using MEGA software (v11, Temple University, Philadelphia, PA, USA, [37]). Fungal Rta1 amino acid sequences were obtained from Fungidb (https://fungidb.org/, accessed on 20 April 2022).

### 2.12. Promoter Analysis

Promoter analysis of 1000 bp of the 5′ untranslated region upstream of the ATG in *RTA1* (using sequence from both *Cn* and *S. cerevisiae*) was analyzed using MEME [38], GOMo [39] and visual inspection with the help of YEASTRACT+ [40].

### 2.13. Real-Time Quantitative PCR

To determine the expression of *RTA1* in other secretion mutants, the HK FOA pKUTAP control strain (a H99 MATα, *ura5* strain transformed with a pORA-KUTAP plasmid without the RNAi construct and the parent strain to the isec6 mutant [11]), *isec6*, *Δsec14-1*, *Δsec14-2* (*Sfh1*), and H99 were resurrected on YNB plates, then grown overnight in YNB 2% glucose at 30 °C with shaking at 250 rpm. Cultures were washed twice in water, then resuspended in 10 mL YNB 2% glucose at 1 OD_600_/mL, incubated at 30 °C with shaking at 250 rpm for 5.5 h. Cultures were snap-frozen and stored at −80 °C.

Total RNA was extracted using TRIzol (Invitrogen, Waltham, MA, USA, catalog #15596026), according to the manufacturer’s instructions. cDNA was prepared using Moloney murine leukemia virus reverse transcriptase (Promega, Madison, WI, USA, catalog #M1701) with Oligo (dT) primers. Gene expression was measured using qPCR with Platinum SYBR green qPCR SuperMix-UDG (Life Technologies, Inc., Carlsbad, CA, USA, catalog #11733038) on the CFX-96 Touch Real-Time PCR Detection System (Bio-Rad Laboratories, Hercules, CA, USA). cDNA reverse transcribed from 200 ng RNA was added per reaction. Fold-change in target gene expression was normalized to actin (*ACT1*) using the 2^−ΔΔCt^ method [41] relative to H99 or to the relevant parent strain (HK for iSec6 mutant; H99 for Sec14 mutants).

### 2.14. Transmission Electron Microscopy (TEM)

TEM was conducted similar to [12], with minor differences. Briefly, the H99, *rta1Δ*, and *rta1Δ+RTA1* strains were grown in YPD to log-phase at 37 °C and then centrifuged. Pellets were fixed in 2% glutaraldehyde (Sigma Alrich, St. Louis, MO, USA, catalog #G5882) in 0.1 M sodium cacodylate buffer under vacuum for 1.5 h, and then incubated in 4% formaldehyde (Sigma Aldrich, St. Louis, MO, USA, catalog #1.00496) and 1% glutaraldehyde in 0.1 M sodium cacodylate buffer for 28 h. Cells were embedded in agar and handled as tissue pieces for the remainder of the fixation. Cells were post fixed in 2% osmium tetroxide (Sigma Aldrich, St. Louis, MO, USA, catalog #75633), followed by 2% uranyl acetate (Fisher Scientific, catalog #18-607-644), and dehydrated through a graded ethanol series. The entire fixation was conducted at room temperature. The fixation and embedding were conducted under vacuum. Ultrathin sections were cut on a Leica Ultracut UCT, stained with lead citrate, and viewed on a Hitachi H-7650 transmission electron microscope.

### 2.15. Crystal Structure of 7-Aminocholesterol

To better understand the Rta1 mechanism of resistance to 7-aminocholesterol, the 7-aminocholesterol compound was synthesized according to a previously reported procedure [42] and crystalized. The crystals for the 7-aminocholesterol imine derivative were prepared by slow evaporation of an acetone solution for 6 days at room temperature and collected by filtration. A Rigaku diffractometer (Tokyo, Japan) with Molybdenum Kα radiation was used for all measurements. Standard crystallography methods were used for structure solution and refinement [43]. A summary of the crystal data and details pertaining to the intensity data collection are given in Table S2. The structure for the 7-aminocholesterol imine derivative was solved using direct methods [44] and refined by the structure factor least squares method using SHELXL-97 [44]. The refined crystal structure generated using Ortep [45] is shown in Figure S8. A summary of the refinement statistics is also in Table S2 and the crystallography information along with the atomic coordinates and structure factors are provided in Table S3.

### 2.16. Statistics

Differences in urease and laccase secretion were analyzed using analysis of variance (ANOVA). One-way ANOVA with Tukey’s multiple comparisons test was used to identify significant differences in real-time qPCR fold-change. Differences in GXM secretion and capsule thickness were analyzed using multivariate analysis of variance (MANOVA) with simple contrasts. Statistical analysis was conducted using JMP, version 14 (SAS Institute, Cary, NC, USA). *p* < 0.05 was considered significant.

## 3. Results

### 3.1. Sequence Homology

**Figure 1:**
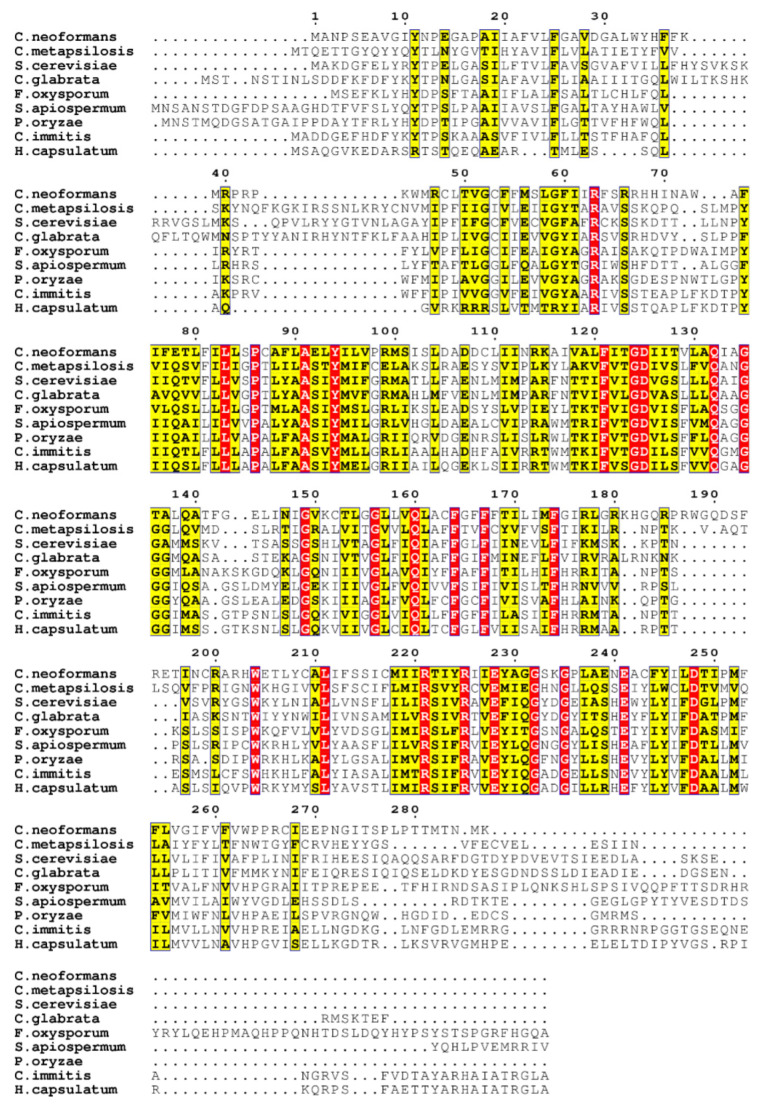
Sequence comparison between fungal Rta1 proteins. ESPript 3.0 [49] was used to highlight sequence similarity (indicated by red boxes).

The sequences of *Cn* Rta1 and its fungal homologues, including *S. cerevisiae* [4], were compared using Clustal Omega (Figure 1, [46,47]). The sequence identity/similarity of *Cn* Rta1 with the other fungal homologues was calculated using Ident and Sim [48]. The sequence identity of *Cn* Rta1 with the other fungal homologues was between 19.4–24.9%, while the sequence similarity was between 34.6–43.2%.

### 3.2. Rta1 Phylogeny

**Figure 2:**
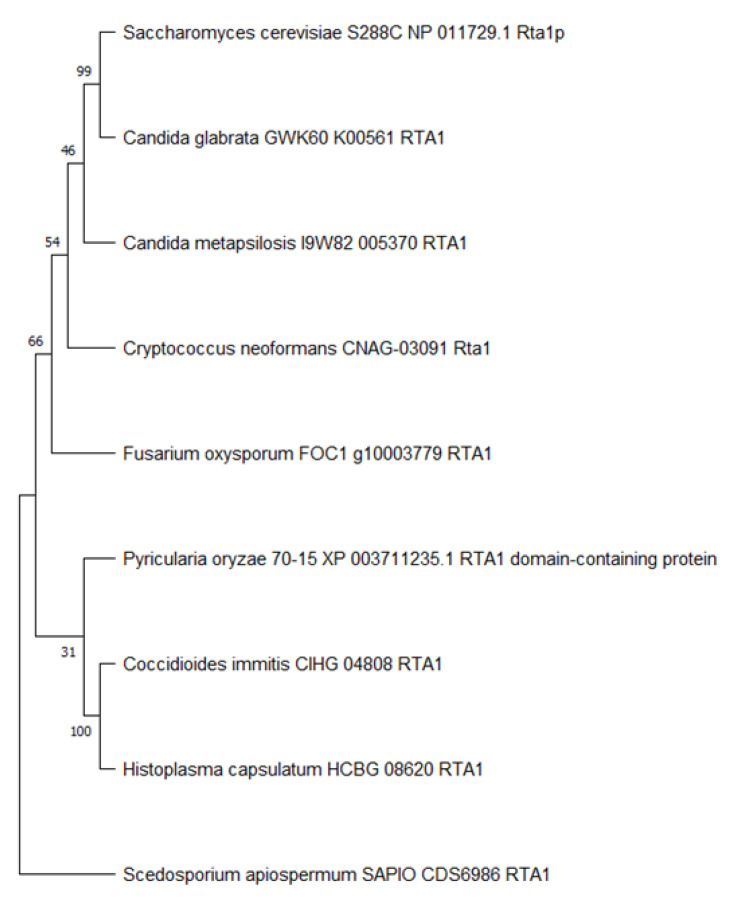
Maximum likelihood phylogenetic tree of fungal Rta1 amino acid sequences after 1000 bootstraps. The phylogenetic tree was created using MEGA software (v11, Temple University, Philadelphia, PA, USA [37]. Numbers on the branches indicate the bootstrap percentage after 1000 replications in constructing the tree.

To better determine the relationship of *Cn* Rta1 to other fungal homologues, a phylogenetic tree of fungal Rta1 amino acid sequences was created. While Rta1 from *S. cerevisiae* was more closely related to Rta1 from *Candida glabrata*, *Cn* Rta1 was equally related to Rta1 from *Candida metapsilosis*, *S. cerevisiae*, and *C. glabrata* (Figure 2). These relationships are concordant with the sequence identity/similarity seen in Figure S4 and the similar phenotype of Rta1 observed in both *S. cerevisiae* and *C. glabrata* [4,50,51].

### 3.3. RTA1 Is Involved in Resistance to 7-Aminocholesterol

**Figure 3:**
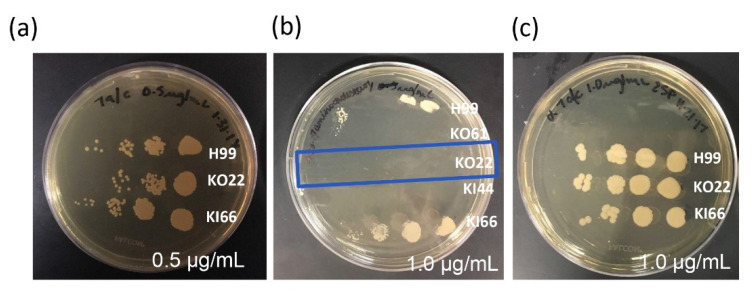
Resistance to 7-aminocholesterol. 7-aminocholesterol was resuspended in dimethyl sulfoxide and then added to YPD agar plates at two concentrations: 0.5 µg/mL (**a**) and 1.0 µg/mL (**b**) or 1.0 µg/mL of the α-7-aminocholesterol derivative was used as a control (**c**). Strains were grown in rich YPD media and 1 × 10^6^ cells/mL were 10-fold diluted and 10 µL of each concentration was spotted onto the plates. There is inhibition of the KO22 (*rta1Δ*) strain, but reduced inhibition of the H99 and KI66 (*rta1Δ+RTA1*) strains. The blue box indicates the *rta1Δ* strain.

To determine if the *Cn RTA1* gene was involved in resistance to 7-aminocholesterol, cells were serially diluted and spotted on YPD plates containing increasing concentrations of 7-aminocholesterol. These data indicated that the *rta1Δ* strain was mildly inhibited at 0.5 µg/mL (Figure 3a) and completely inhibited at 1.0 µg/mL 7-aminocholesterol (Figure 3b), while the H99 and *rta1Δ+RTA1* strains both showed normal growth. As a control, strains were also plated on a derivative of 7-aminocholesterol (α-7-aminocholesterol) with no activity (Figure 3c).

### 3.4. Intracellular 7-Aminocholesterol Visualization

**Figure 4:**
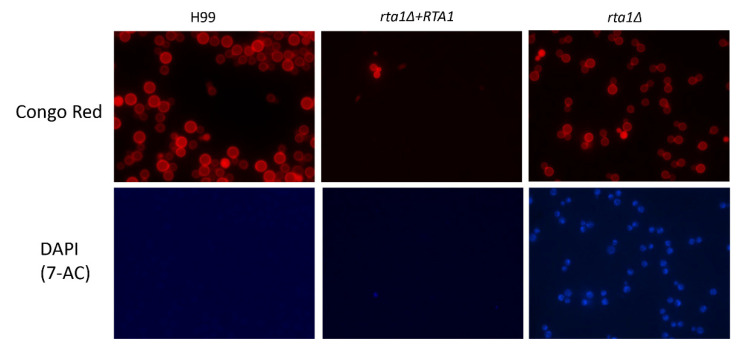
Fluorescent 7-aminocholesterol (7-AC) is trapped within the cell in the *rta1Δ* strain. Cells were visualized using fluorescence microscopy after overnight growth in the presence of fluorescent dansyl 7-aminocholesterol and subsequent staining with Congo red (to visualize the cell wall). The *rta1Δ+RTA1* strain was inadvertently started with a smaller than normal frozen innocula in this experiment, so has few cells. All images were taken using the same exposure time.

Since *RTA1* appeared to be involved in resistance to 7-aminocholesterol, possibly by transporting 7-aminocholesterol out of the cell, the H99, *rta1Δ*, and *rta1Δ+RTA1* strains were grown in the presence of fluorescent dansyl 7-aminocholesterol overnight and then stained with Congo red to visualize the cell wall and determine if we could visualize 7-aminocholesterol trapped within the cell of the *rta1Δ* strain. While fluorescent microscopy revealed specific punctate spots within the *rta1Δ* strain using the DAPI filter (which captures dansyl fluorescence), there was no specific DAPI staining in the H99 and *rta1Δ+RTA1* strains (Figure 4).

### 3.5. Secretion Phenotype of the H99, rta1Δ, and rta1Δ+RTA1 Strains

**Figure 5:**
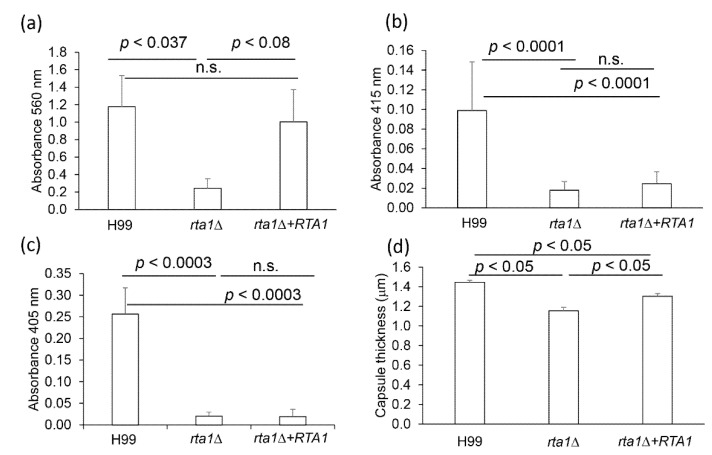
(**a**) Urease secretion. Error is standard error of the mean. N = 5. (**b**) Laccase secretion. Error is standard error of the mean. N = 4. (**c**) Glucuronoxylomannan (GXM) secretion. Error is standard error of the mean. N = 4. (**d**) Capsule thickness. Capsule thickness was measured on >70 cells per strain using Zeiss Axiovision software v.4.9.1 on a Zeiss A1 inverted microscope (100× magnification). Capsule thickness was calculated by subtracting the cell body diameter from the diameter of the entire cell + capsule and dividing by 2. Error is standard error of the mean. N = 3.

Because *RTA1* was involved in resistance to 7-aminocholesterol, possibly by transporting it out of the cell, we hypothesized that *RTA1* may also be involved in exocystic secretion. Thus, the H99, *rta1Δ*, and *rta1Δ+RTA1* strains were phenotyped for virulence factors known to be secreted, including phospholipase B, urease, laccase, and GXM [12,19]. While there was no difference between the *rta1Δ* strain and the H99 strain in phospholipase secretion (Figure S5) or melanin production (Figure S6), the *rta1Δ* strain secreted significantly less urease than the H99 strain (*p* = 0.037) and showed a trend for less secretion as compared to the *rta1Δ+RTA1* strain (*p* = 0.08) (Figure 5a). Additionally, laccase (*p* < 0.0001, for both strains, Figure 5b) and GXM secretion (*p* < 0.0003, for both strains, Figure 5c) were significantly lower in the *rta1Δ* strain compared to that in H99. In accordance with the GXM secretion data, the *rta1Δ* strain had a significantly smaller capsule thickness compared to both H99 and the *rta1Δ+RTA1* strain (*p* < 0.05 for both comparisons), while the *rta1Δ+RTA1* strain had a significantly larger capsule as compared to the *rta1Δ* strain (*p* < 0.05) (Figure 5d). These data suggested that *RTA1* may be involved in secretion in *Cn*.

### 3.6. Urease Secretion in Different Secretion Mutants

Secretion of urease, laccase, and GXM in *Cn* occurs via the Sec6 pathway [11] and through extracellular vesicles [19], while optimal secretion of phospholipase B and acid phosphatase (Aph1) via non endosome- and endosome-dependent routes, respectively, depends upon a functional Sec14 pathway [16]. The Sec14 pathway is not required for laccase or GXM secretion [52], but the role of the Sec14 pathway in urease secretion in *Cn* was never investigated. Thus, urease secretion was tested in the H99, *sec14Δ* and Sfh1 (Sec14-2) strains. Sec14-2 is a homologue of Sec14, but has an insignificant role in Sec14-dependent secretory processes and pathogenicity [16]. No difference was found between H99 and *sec14Δ* and only a small reduction in urease secretion was observed in the *sec14-2*Δ strain (*sfh1Δ*, Figure S7).

### 3.7. qPCR of RTA1 Expression in Different Secretion Mutants

**Figure 6:**
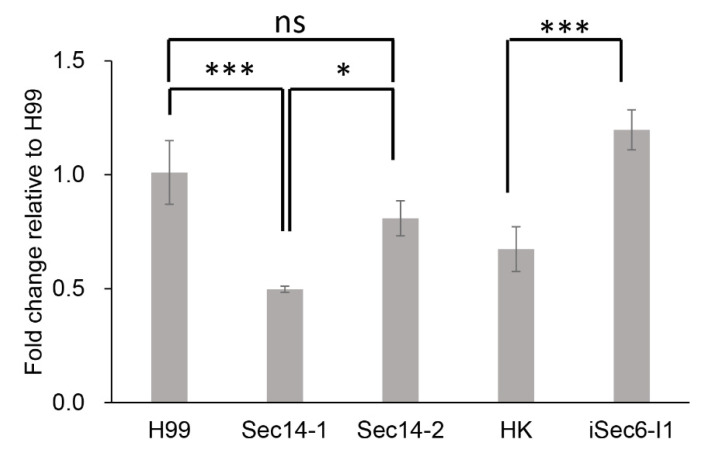
Real-time qPCR of *RTA1* expression in other secretion mutants. In contrast to the Sec14-1 mutant, the Sec14-2 mutant (*Sfh1*) has an insignificant role in Sec14-dependent secretory processes. HK is the parent strain of the RNAi *iSec6* mutant in which approximately 50% suppression of Sec6 has been achieved [11]. * *p* < 0.05, *** *p* < 0.001.

Because secretion of urease, laccase, and GXM in *Cn* occurs via the Sec6 pathway and phospholipase secretion occurs via the Sec14 pathway, *RTA1* expression was determined in the H99 wild-type strain, and a number of secretion mutants, including *sec14-1Δ*, *Sfh1* (Sec14-2), and the iSec6 mutant (a kind gift of Peter Williamson) via real-time qPCR. *RTA1* expression in *Sec14-1Δ* is approximately 2-fold lower than that in H99, while there is a small reduction in *RTA1* expression in *Sec14-2Δ* relative to H99 (Figure 6). In contrast, *RTA1* expression in the *iSec6-I1* mutant is approximately double that of its HK parent strain (Figure 6). These data suggested that *RTA1* may have links with both the Sec14 and Sec6 secretion pathways.

### 3.8. TEM of the H99, rta1Δ, and rta1Δ+RTA1 Strains

**Figure 7:**
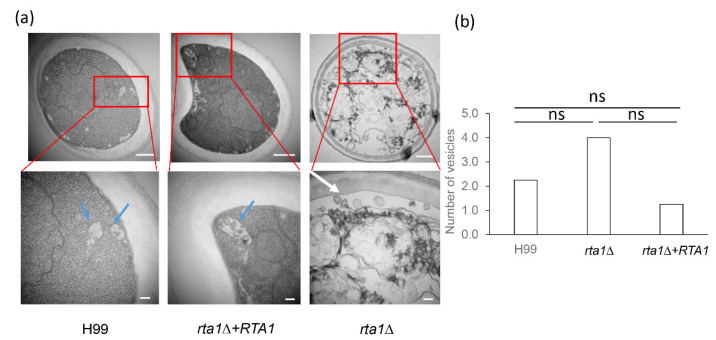
Transmission electron microscopy images of H99, *rta1Δ+RTA1* and *rta1Δ* (**a**). The *rta1Δ* strain has an accumulation of vesicles under the cell membrane (indicated by white arrows), while H99 and the *rta1Δ+RTA1* strain do not. The bottom row of images is a magnification of the images above. Scale bars in the top row are 500 nm and scale bars in the bottom row are 100 nm. The structures in H99 and *rta1Δ+RTA1* indicated with blue arrows appear to be multivesicular bodies with internalized exosomes ready to be secreted. (**b**) Bar graph depicting the number of vesicles counted for four images for each strain. ns, not significant.

To look for possible secretion defects in the *rta1Δ* strain, TEM was performed to resolve secretory vesicles. The results demonstrated an accumulation of vesicles in the *rta1Δ* strain, but not in the H99 and *rta1Δ+RTA1* strains (Figure 7). These vesicles had an approximate size of <100 nm and were predominantly found near the plasma membrane.

### 3.9. Potential Model of Rta1 Strucure in the Membrane

**Figure 8:**
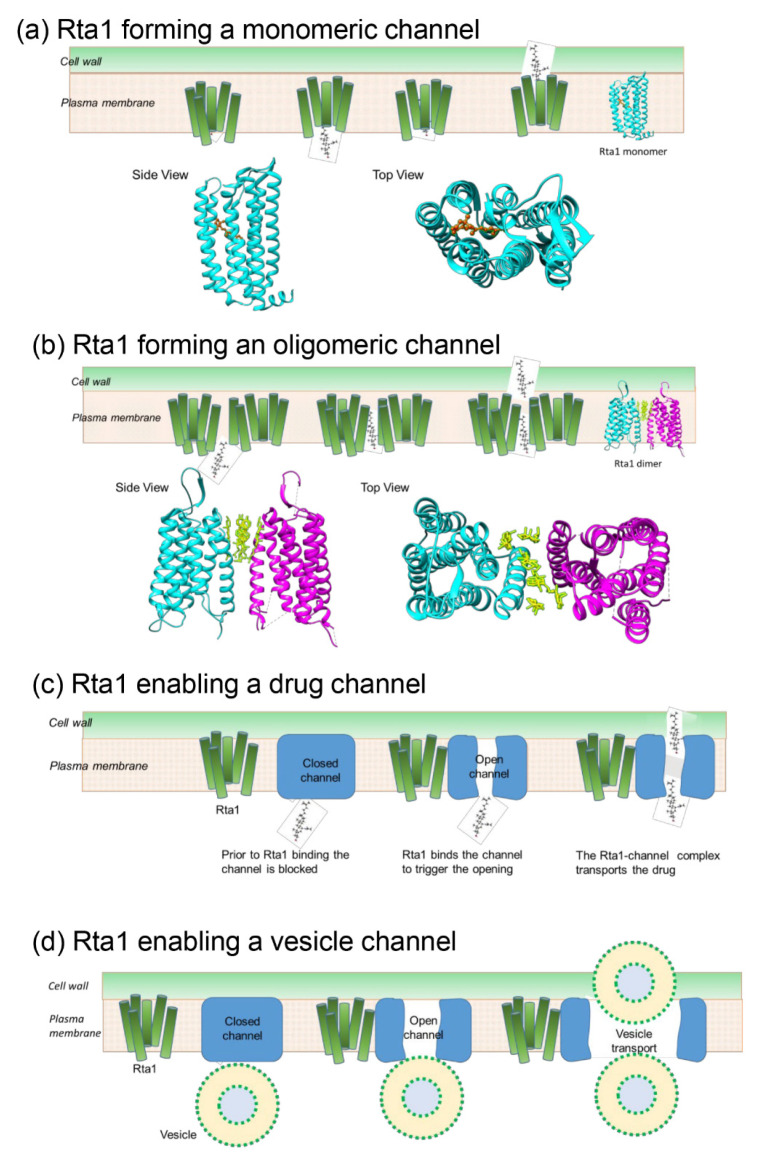
Rta1 may be involved in both vesicle-dependent secretion and transport of 7-aminocholesterol in *C. neoformans*. Possible models of Rta1 that would allow export of 7-aminocholesterol or vesicles. (**a**) Monomeric model. The CIR protein (PDB code: 5G28) is used as a candidate for modeling Rta1 due to its similar size in helices, loops, and total amino acid count. The protein, shown in a cyan ribbon representation, can be seen from the top and side views. In addition, it is a 7 alpha helical bundle, as Rta1 is predicted to be. Chimera modeling based on the amino acid sequence and known helical sequence indicate that CIR also binds molecules (yellow stick model) of a similar size to 7-aminocholesterol. (**b**) Oligomeric Rta1 may form a channel that may be large enough to transport vesicles. The rhodopsin dimer (PDB code: 4OR2) is used to generate this model to illustrate a potential channel at the dimer interface. The monomers are distinguished in cyan and magenta while the drug is shown as a yellow stick model (**c**) Rta1 may bind to a channel (blue rectangle) triggering the channel opening to allow export of drugs. (**d**) A schematic of Rta1 enabling a channel to allow export of vesicles.

Rta1 in *S. cerevisiae* is predicted to be a protein with seven transmembrane helices [4]. Secondary structure predictions generated by Quick 2D and PsiPred indicated that *Cn* Rta1 also had seven helices (Figure S4). Because crystallization of transmembrane proteins is challenging, homology modeling was used to determine if there was a structural rationale to explain the involvement of Rta1 in secretion. Although a crystal structure of Rta1 would give high-resolution information of its structure, the models predicted here, nonetheless, correlate well with the experimental data. A three-dimensional model for *Cn* Rta1 was generated, as described in the methods section, and compared with known protein structures having similar topology (Table 1) to determine if Rta1 forms a channel in the plasma membrane that could transport 7-aminocholesterol or secretory vesicles out of the cell. Three possible models of Rta1 were generated based on the protein’s oligomeric state to explain the secretion of 7-aminocholestrol (Figure 8a–c). A model for Rta1-mediated vesicle secretion is shown in Figure 8d.

In the first model, Rta1, a predicted seven-helix bundle, may function as a monomer (Figure 8a). The monomeric structure contains a pore through the center of the 7-helix bundle that may act as a channel to export a ligand, such as an extended molecule like 7-aminocholesterol (Figure S8). The light-driven chloride pump CIR (PDB: 5G28) exhibited a similar secondary structure to Rta1 with a transmembrane domain consisting of seven alpha helices. Notably, CIR acts to pump small molecules across the plasma membrane [36], which is similar to the hypothesized function of Rta1 transporting 7-aminocholesterol out of the cell. The helical structure of the entire CIR protein derived using Chimera, suggested that a molecule of approximately the same size and with a similar structure to 7-aminocholesterol (Figure S8) could fit through the predicted monomeric channel. In the second model, Rta1 may function as an oligomer (Figure 8b). Structural homologs of the CIR protein, such as human class C G protein-coupled metabotropic glutamate receptor 1 (PDB 4OR2, Table 1), were used to generate oligomeric models of Rta1, which revealed the possibility of channels being formed at the dimeric- or tetrameric-interfaces. If oligomeric Rta1 forms a channel, the channel may be large enough to allow export of secretory vesicles or drugs (Figure 8b). In the third model, Rta1 may be a catalyst to open or close an unknown channel (Figure 8c), which would allow export of the drug. A similar Rta1-mediated pathway may result in the export of secretory vesicles (Figure 8d).

## 4. Discussion

*RTA1* (TC.9.A.26.1.1) in *S. cerevisiae* confers resistance to 7-aminocholesterol when overexpressed [4]. In addition to *RTA1*, another potential homolog in *S. cerevisiae*, *RSB1* (TC 9.A.26.1.2), protects the cell from cell toxicity through prevention of exogenous phytosphingosine (PHS) accumulation [53]. Since the homologs in the literature suggested that the *S. cerevisiae* homologs conferred resistance to 7-aminocholesterol or PHS build-up, both 7-aminocholesterol and PHS build-up were tested. Only a difference in resistance to 7-aminocholesterol, which is thought to act like a morpholine drug class and inhibit the ergosterol biosynthesis pathway [6], was observed in *Cn*.

Since the *RTA1* mutants in *Cn* and *S. cerevisiae* shared phenotypic similarity, amino acid similarity was calculated to obtain an indication of whether their three-dimensional architectures were also similar. However, the amino acid sequences were less than 45% identical or similar. This trend was also observed in the phylogenetic analysis where *Cn* Rta1 was equally similar to Rta1 from *Candida metapsilosis*, *S. cerevisiae*, and *C. glabrata* while *S. cerevisiae* Rta1 was more similar to Rta1 from *C. glabrata*. These data are in agreement with the data of Kołaczkowska et al. showing a similar phenotype of *C. glabrata RTA1* and *S. cerevisiae RTA1*. *C. glabrata RTA1* confers resistance to 7-aminocholesterol when overexpressed [51], similar to *S. cerevisiae RTA1*, and its expression is also correlated with drug resistance to azoles and conditions inducing ergosterol depletion, such as hypoxia [50], also similar to *S. cerevisiae*. To determine if *Cn RTA1* might also be regulated by hypoxia and ergosterol deletion, similar to that of *RTA1* in *C. glabrata*, promoter analysis of *Cn RTA1* was conducted. Similar binding motifs for transcription factors that regulate responses to hypoxia and disruptions in ergosterol biosynthesis that have been previously identified in the promoter of *S. cerevisiae RTA1* were observed, including Mot3p, Rox1p, and Upc2 [50]. Future experiments will test the hypothesis that *Cn RTA1* may also be regulated by hypoxic conditions or ergosterol deletion.

As observed in *S. cerevisiae*, disruption of the *RTA1* gene showed decreased resistance to 7-aminocholesterol [4]. Concentrations at which inhibition were observed in *Cn* were also consistent with results in *S. cerevisiae*. In *S. cerevisiae*, *RTA1* disruption leads to resistance at <2.5 µM (1.0 µg/mL) [4], which is consistent with data of this study. Fluorescence microscopy with a fluorescent 7-aminocholesterol clearly showed that the drug was trapped within the cell in the *rta1Δ* strain, but not within the cells of the H99 or *rta1Δ+RTA1* strains. Together these data suggest that *RTA1* is responsible for transporting 7-aminochoesterol out of the cell. Accordingly, deletion of *RTA1* in the *rta1Δ* mutant led to accumulation of 7-aminocholesterol and cell death.

Interestingly, while the *rta1Δ+RTA1* strain recapitulated the wild-type phenotype of resistance to 7-aminocholesterol and urease secretion, it did not rescue the wild-type secretion phenotype for laccase and GXM. We are not sure why this was the case, but hypothesize that the incorporation of the *rta1Δ+RTA1* construct in the reconstituted strain may have disrupted conventional secretion, as both laccase and GXM are also secreted via the conventional secretion pathway [52].

In this study, *RTA1* was initially identified in a screen for mutants that did not produce melanin, which is produced by the secreted virulence factor laccase [11]. Interestingly, when the *rta1Δ* strain was created, no difference in melanin production or pigmentation was observed in comparison to the wildtype H99 strain. However, laccase production in this mutant was tested after reductions in urease and GXM were identified in the *rta1Δ* strain to determine in which secretion pathways *RTA1* might play a role. Due to the differences in secretion of laccase, urease, and GXM in the *rta1Δ* strain, but no difference in phospholipase secretion in comparison to the H99 strain, we hypothesized that *RTA1* was part of the Sec6 secretion pathway, which is involved in the secretion of extracellular vesicles. Thus, *RTA1* expression was tested in the iSec6 and *sec14Δ* mutant strains. Unexpectedly, *RTA1* expression was reduced in the *sec14-1Δ* mutant and increased in the iSec6 mutant, suggesting that Rta1 was connected to both secretion pathways. The reduction of *RTA1* in the *sec14-1Δ* mutant fits with the lack of a difference in phospholipase B secretion observed in the *rta1Δ* strain because phospholipase B was measured during growth on YPD and Sec14-dependent differences in phospholipase B have only been observed when grown on YNB [16]. Since YPD is a rich medium and YNB is a minimal medium there will be changes in nutrient ratios that effect all aspects of metabolism. Thus, it is likely that secretion and processing of GPI anchors (including that associated with phospholipase) would also be affected. Future experiments will test phospholipase secretion in the *rta1Δ* strain when grown in YNB.

**Figure 9:**
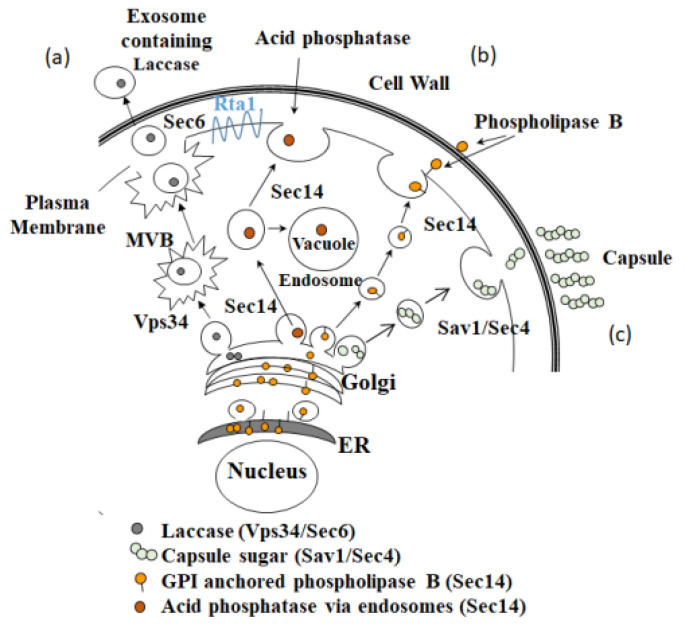
Known secretion pathways and hypothesized model of Rta1 location in *C. neoformans*. (**a**) The Sec6-mediated secretion pathway. Proteins, including laccase and soluble polysaccharide [11], are packaged in the Golgi and move toward the cell membrane via multivesicular bodies (MVB) [11,52]. (**b**) The Sec14-mediated pathway. Sec14p is a non-enzymatic phosphatidylinositol (PI) transfer protein. It serves as a lipid sensor to coordinate phosphatidylcholine (PC) and PI 4-phosphate (PI4P) metabolism to establish appropriate PI4P levels for maintaining Golgi integrity and regulating secretory vesicle biogenesis [55]. Sec14-dependent post-Golgi secretory vesicles transport phospholipase B directly to the periphery [16]. They also transport acid phosphatase to the periphery or vacuoles via larger secretory structures called endosomes [17]. (**c**) The Sav1/Sec4-mediated pathway. Soluble polysaccharides are also transported to the fungal cell periphery via post-Golgi secretory vesicles [13,52].

Interestingly, in the RNAi iSec6 mutant that only had ~50% Sec6 expression compared to its parent HK strain, *RTA1* expression was increased suggesting that increased *RTA1* expression may compensate for the reduced Sec6 expression in order to maintain appropriate secretion levels in this mutant. These data suggest that Rta1, as a potential lipid transporter, may be involved in both the Sec6 non-conventional secretion pathway and conventional Sec14 secretion via non endosome- and endosome-dependent routes (Figure 9). Additionally, since GXM has been shown to be secreted via multiple secretion pathways, including Sec6 [11], Sav1/Sec4 [13], and via Golgi reassembly and stacking proteins (GRASPs) [54], and the *rta1Δ* strain had a significantly smaller capsule, *RTA1* may also be involved the Sav1/Sec4 secretion pathway. Future experiments will test this hypothesis.

One of the biggest unresolved questions concerning *Cn* secretion is how a class of secretory vesicles known as exosomes are transported across the plasma membrane and cell wall. Given that Rta1 is required for the secretion of exosome cargo and is potentially a lipid transporter, Rta1 could function as a gatekeeper for transporting exosomes out of the cell. Although the vesicles observed to accumulate in the *rta1Δ* mutant are smaller (< 100 nm) than those of exosomes (100–300 nm), they are not contained within multivesicular bodies. Thus, further studies are needed to confirm whether these vesicles are exosomes or another type of secretory vesicle. However, given the secretory vesicle accumulation observed in the *Sec6* [11] and *Sav1* mutants [13], the size of the vesicles accumulating in the *rta1Δ* mutant, and the virulence factor secretion defect observed in this strain, we believe these accumulated vesicles are, in fact, secretory vesicles.

In conclusion, these data suggest that Rta1 is a seven-helix transmembrane protein that confers resistance to 7-aminocholesterol, possibly by directly transporting the drug out of the cell and that Rta1 is involved in both conventional and non-conventional secretion of virulence factors. Since urease, laccase, and GXM can be exported from the cell in vesicles [9,16] and Rta1 likely belongs to a lipid-translocating exporter family, Rta1 may be involved in the export of these vesicles across the cell membrane. Future studies are needed to definitively test this hypothesis. Additionally, since Rta1 is not found in plants and animals, it represents a strategic target for antifungal drug development.

## Figures and Tables

**Table 1:** List of proteins from the protein data bank (PDB) that also had seven transmembrane regions, similar predicted gene ontologies, such as pores or channels, and were of similar size to Rta1 (TC # 9.A.26.1.1). The transporter classification (TC) number is also indicated.

PDB ID	TC #	Description
4OR2	9.A.14.7.1	Human class C G protein-coupled metabotropic glutamate receptor 1 in complex with a negative allosteric modulator
3RKO	3.D.1.1.1	Crystal structure of the membrane domain of respiratory complex I from *E. coli* at 3.0 angstrom resolution
3AG3	3.D.4.7.1	Bovine heart cytochrome C oxidase in the nitric oxide-bound fully reduced state at 100 K
5G28	3.E.1.6.14	The crystal structure of light-driven chloride pump ClR at pH 6.0
4HYJ	3.E.1.6.5	Crystal structure of *Exiguobacterium sibiricum* rhodopsin
3EML	9.A.14.3.8	The 2.6 A crystal structure of a human A2A adenosine receptor bound to ZM241385

## Data Availability

The data presented in this study are available within the article, supplementary material, and at the protein data bank (www.pdb.org).

## Supplementary Materials

The following are available online at https://www.mdpi.com/article/10.3390/pathogens11111239/s1, Figure S1: Knockout mutants that did not make melanin, Figure S2: Restriction maps of the *rta1Δ* and *rta1Δ+RTA1* constructs, Figure S3: Confirmation of *rta1Δ+RTA1* construct insertion into *C. neoformans* using PCR and Southern blot analysis, Figure S4: Secondary structure protein modeling for Rta1, Figure S5: Phospholipase secretion in H99 and *rta1Δ*. Figure S6: Melanin production in H99 and *rta1Δ*. Figure S7: Urease is not secreted via the Sec14 pathway, Figure S8: Perspective view of the crystal structure of 7-aminocholesterol generated using Ortep-III, Table S1: List of primers used for amplification and diagnostics of *RTA1*, Table S2: Crystal data, details of data collection, and refinement parameters for 7-aminocholesterol, Table S3: Crystal structure collection data for 7-aminocholesterol.

## References

[1] Heitman J., Kozel T.R., Kwon-Chung J., Perfect J.R., Casadevall A. (2011). Cryptococcus: From Human Pathogen to Model Yeast.

[2] Rajasingham R., Smith R.M., Park B.J., Jarvis J.N., Govender N.P., Chiller T.M., Denning D.W., Loyse A., Boulware D.R. (2017). Global burden of disease of HIV-associated cryptococcal meningitis: An updated analysis. Lancet Infect. Dis..

[3] McClelland E.E., Casadevall A., Eisenman H.C., Kavanagh K. (2007). Pathogenesis of *Cryptococcus neoformans*. New Insights in Medical Mycology.

[4] Soustre I., Letourneux Y., Karst F. (1996). Characterization of the *Saccharomyces cerevisiae RTA1* gene involved in 7-aminocholesterol resistance. Curr. Genet..

[5] Alhanout K., Djouhri L., Vidal N., Brunel J.M., Piarroux R., Ranque S. (2011). In Vitro activity of aminosterols against yeasts involved in blood stream infections. Med. Mycol..

[6] Elkihel L., Soustre I., Karst F., Letourneux Y. (1994). Amino- and aminomethylcholesterol derivatives with fungicidal activity. FEMS Microbiol. Lett..

[7] Lorenz R.T., Parks L.W. (1992). Cloning, sequencing, and disruption of the gene encoding sterol C-14 reductase in *Saccharomyces cerevisiae*. DNA Cell Biol..

[8] Ashman W.H., Barbuch R.J., Ulbright C.E., Jarrett H.W., Bard M. (1991). Cloning and disruption of the yeast C-8 sterol isomerase gene. Lipids.

[9] Weete J.D. (1989). Structure and function of sterols in fungi. Adv. Lipid Res..

[10] TerBush D.R., Novick P. (1995). Sec6, Sec8, and Sec15 are components of a multisubunit complex which localizes to small bud tips in *Saccharomyces cerevisiae*. J. Cell Biol..

[11] Panepinto J., Komperda K., Frases S., Park Y.D., Djordjevic J.T., Casadevall A., Williamson P.R. (2009). Sec6-dependent sorting of fungal extracellular exosomes and laccase of *Cryptococcus neoformans*. Mol. Microbiol..

[12] Rodrigues M.L., Nimrichter L., Oliveira D.L., Frases S., Miranda K., Zaragoza O., Alvarez M., Nakouzi A., Feldmesser M., Casadevall A. (2007). Vesicular polysaccharide export in *Cryptococcus neoformans* is a eukaryotic solution to the problem of fungal trans-cell wall transport. Eukaryot. Cell.

[13] Yoneda A., Doering T.L. (2006). A eukaryotic capsular polysaccharide is synthesized intracellularly and secreted via exocytosis. Mol. Biol. Cell.

[14] Salminen A., Novick P.J. (1987). A ras-like protein is required for a post-Golgi event in yeast secretion. Cell.

[15] Curwin A.J., Fairn G.D., McMaster C.R. (2009). Phospholipid transfer protein Sec14 is required for trafficking from endosomes and regulates distinct trans-Golgi export pathways. J. Biol. Chem..

[16] Chayakulkeeree M., Johnston S.A., Oei J.B., Lev S., Williamson P.R., Wilson C.F., Zuo X., Leal A.L., Vainstein M.H., Meyer W. (2011). SEC14 is a specific requirement for secretion of phospholipase B1 and pathogenicity of *Cryptococcus neoformans*. Mol. Microbiol..

[17] Lev S., Crossett B., Cha S.Y., Desmarini D., Li C., Chayakulkeeree M., Wilson C.F., Williamson P.R., Sorrell T.C., Djordjevic J.T. (2014). Identification of Aph1, a phosphate-regulated, secreted, and vacuolar acid phosphatase in *Cryptococcus neoformans*. mBio.

[18] Schekman R. (2002). Lasker Basic Medical Research Award. SEC mutants and the secretory apparatus. Nat. Med..

[19] Rodrigues M.L., Nakayasu E.S., Oliveira D.L., Nimrichter L., Nosanchuk J.D., Almeida I.C., Casadevall A. (2008). Extracellular vesicles produced by *Cryptococcus neoformans* contain protein components associated with virulence. Eukaryot. Cell.

[20] Charlier C., Chretien F., Baudrimont M., Mordelet E., Lortholary O., Dromer F. (2005). Capsule structure changes associated with *Cryptococcus neoformans* crossing of the blood-brain barrier. Am. J. Pathol..

[21] Granger D.L., Perfect J.R., Durack D.T. (1985). Virulence of *Cryptococcus neoformans*. Regulation of capsule synthesis by carbon dioxide. J. Clin. Investig..

[22] Casadevall A., Mukherjee J., Scharff M.D. (1992). Monoclonal antibody based ELISAs for cryptococcal polysaccharide. J. Immunol. Methods.

[23] Eisenman H.C., Chow S.K., Tse K.K., McClelland E.E., Casadevall A. (2011). The effect of L-DOPA on *Cryptococcus neoformans* growth and gene expression. Virulence.

[24] Kwon-Chung K.J., Wickes B.L., Booth J.L., Vishniac H.S., Bennett J.E. (1987). Urease inhibition by EDTA in the two varieties of *Cryptococcus neoformans*. Infect. Immun..

[25] Zhang S., Varma A., Williamson P.R. (1999). The yeast *Cryptococcus neoformans* uses ‘mammalian’ enhancer sites in the regulation of the virulence gene, CNLAC1. Gene.

[26] Casadevall A., Perfect J.R. (1998). Cryptococcus neoformans.

[27] Zimmermann L., Stephens A., Nam S.Z., Rau D., Kubler J., Lozajic M., Gabler F., Soding J., Lupas A.N., Alva V. (2018). A completely reimplemented MPI bioinformatics toolkit with a new HHpred server at its core. J. Mol. Biol..

[28] Buchan D.W., Minneci F., Nugent T.C., Bryson K., Jones D.T. (2013). Scalable web services for the PSIPRED protein analysis workbench. Nucleic Acids Res..

[29] Mol A.R., Castro M.S., Fontes W. (2018). NetWheels: A Web Application to Create High Quality Peptide Helical Wheel and Net Projections.

[30] Rost B., Yachdav G., Liu J. (2004). The PredictProtein server. Nucleic Acids Res..

[31] Consonni S.V., Maurice M.M., Bos J.L. (2014). DEP domains: Structurally similar but functionally different. Nat. Rev. Mol. Cell Biol..

[32] Petrey D., Fischer M., Honig B. (2009). Structural relationships among proteins with different global topologies and their implications for function annotation strategies. Proc. Natl. Acad. Sci. USA.

[33] Valencia A., Kjeldgaard M., Pai E.F., Sander C. (1991). GTPase domains of ras p21 oncogene protein and elongation factor Tu: Analysis of three-dimensional structures, sequence families, and functional sites. Proc. Natl. Acad. Sci. USA.

[34] Manente M., Ghislain M. (2009). The lipid-translocating exporter family and membrane phospholipid homeostasis in yeast. FEMS Yeast Res..

[35] Pettersen E.F., Goddard T.D., Huang C.C., Couch G.S., Greenblatt D.M., Meng E.C., Ferrin T.E. (2004). UCSF Chimera--a visualization system for exploratory research and analysis. J. Comput. Chem..

[36] Kim K., Kwon S.K., Jun S.H., Cha J.S., Kim H., Lee W., Kim J.F., Cho H.S. (2016). Crystal structure and functional characterization of a light-driven chloride pump having an NTQ motif. Nat. Commun..

[37] Tamura K., Stecher G., Kumar S. (2021). MEGA11: Molecular Evolutionary Genetics Analysis Version 11. Mol. Biol. Evol..

[38] Bailey T.L., Elkan C. (1994). Fitting a mixture model by expectation maximization to discover motifs in biopolymers. Proc. Int. Conf. Intell. Syst. Mol. Biol..

[39] Buske F.A., Boden M., Bauer D.C., Bailey T.L. (2010). Assigning roles to DNA regulatory motifs using comparative genomics. Bioinformatics.

[40] Monteiro P.T., Oliveira J., Pais P., Antunes M., Palma M., Cavalheiro M., Galocha M., Godinho C.P., Martins L.C., Bourbon N. (2020). YEASTRACT+: A portal for cross-species comparative genomics of transcription regulation in yeasts. Nucleic Acids Res..

[41] Livak K.J., Schmittgen T.D. (2001). Analysis of relative gene expression data using real-time quantitative PCR and the 2 (-delta delta C(T)) method. Methods.

[42] El Kihel L., Choucair B., Dhernomez M., Letourneux Y. (2002). Stereoselective synthesis of 7α- and 7β-aminocholesterol as Δ8-Δ7 sterol isomerase inhibitors, with fungicidal activities towards resistant strains. Eur. J. Org. Chem..

[43] Prabu M.M., Nagendra H.G., Suresh S., Vijayan M. (1996). X-ray studies on crystalline complexes involving amino acids and peptides. XXXI. Effect of chirality on ionization state, stoichiometry and aggregation in the complexes of oxalic acid with L- and DL-histidine. J. Biomol. Struct. Dyn..

[44] Sheldrick G.M. (2008). A short history of SHELX. Acta Crystallogr. A.

[45] Farrugia L.J. (1997). ORTEP-3 for Windows—A version of ORTEPIII with a Graphical user Interface (GUI). J. Appl. Cryst..

[46] Madeira F., Pearce M., Tivey A.R.N., Basutkar P., Lee J., Edbali O., Madhusoodanan N., Kolesnikov A., Lopez R. (2022). Search and sequence analysis tools services from EMBL-EBI in 2022. Nucleic Acids Res..

[47] Rice P., Longden I., Bleasby A. (2000). EMBOSS: The European Molecular Biology Open Software Suite. Trends Genet..

[48] Stothard P. (2000). The sequence manipulation suite: JavaScript programs for analyzing and formatting protein and DNA sequences. Biotechniques.

[49] Robert X., Gouet P. (2014). Deciphering key features in protein structures with the new ENDscript server. Nucleic Acids Res..

[50] Kolaczkowska A., Manente M., Kolaczkowski M., Laba J., Ghislain M., Wawrzycka D. (2012). The regulatory inputs controlling pleiotropic drug resistance and hypoxic response in yeast converge at the promoter of the aminocholesterol resistance gene RTA1. FEMS Yeast Res..

[51] Kolaczkowska A., Dylag M., Kolaczkowski M. (2013). Differential expression of the *Candida glabrata* CgRTA1 and CgRSB1 genes in response to various stress conditions. Biochem. Biophys. Res. Commun..

[52] Rodrigues M.L., Djordjevic J.T. (2012). Unravelling secretion in *Cryptococcus neoformans*: More than one way to skin a cat. Mycopathologia.

[53] Skrzypek M.S., Nagiec M.M., Lester R.L., Dickson R.C. (1998). Inhibition of amino acid transport by sphingoid long chain bases in *Saccharomyces cerevisiae*. J. Biol. Chem..

[54] Kmetzsch L., Joffe L.S., Staats C.C., de Oliveira D.L., Fonseca F.L., Cordero R.J., Casadevall A., Nimrichter L., Schrank A., Vainstein M.H. (2011). Role for Golgi reassembly and stacking protein (GRASP) in polysaccharide secretion and fungal virulence. Mol. Microbiol..

[55] Djordjevic J.T., Lev S. (2018). Fungal secretion: The next-gen target of antifungal agents?. Cell Chem. Biol..

