# Supplementary material for: RTA1 Is Involved in Resistance to 7-Aminocholesterol and Secretion of Fungal Proteins in Cryptococcus neoformans
Source: Pathogens. 2022 Oct 26;11(11):1239. doi: 10.3390/pathogens11111239 (PMC9697666; doi:10.3390/pathogens11111239)
Supplement: Supplementary file 1 [file pathogens-11-01239-s001.zip › Supplementary material.pdf]

RTA1 Is Involved in Resistance to 7-Aminocholesterol and Secretion of Fungal Proteins in *Cryptococcus neoformans*

Emily S. Smith-Peavler [1], Ronakkumar Patel [2], Adejumoke Mary Onumajuru [1], Bethany G. Bowring [3], Joyce L. Miller [4], Jean Michel Brunel [5], Julianne T. Djordjevic [3,6], Moses M. Prabu [7] and Erin E. McClelland [8,*]

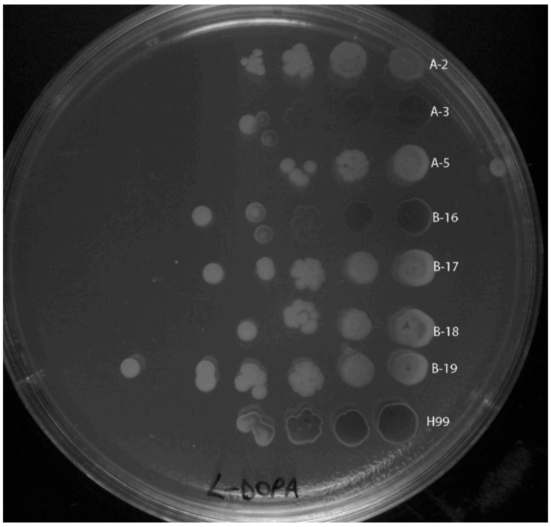

Figure S1. Knockout mutants that did not make melanin. Inverse PCR off the noursethricin gene in the genome of three of the five melanin mutants identified the gene Resistance to aminocholesterol 1 (*RTA1*) as the gene that was disrupted.

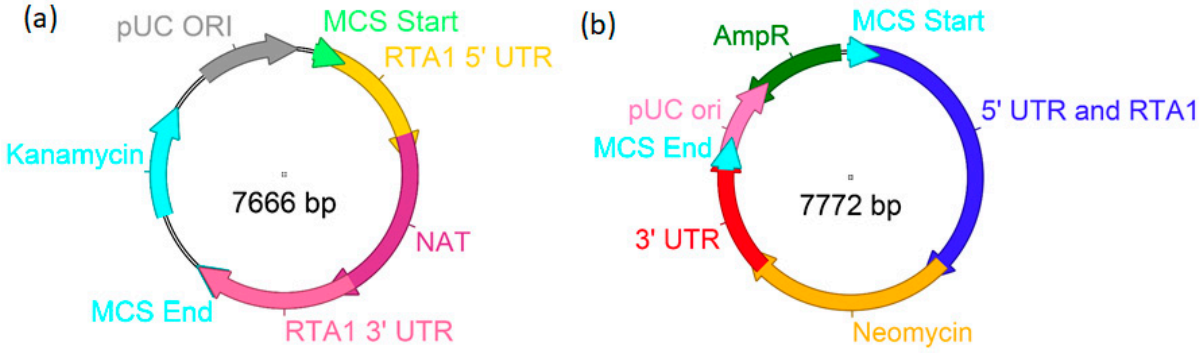

Figure S2. Restriction maps of the *rta1Δ* and *rta1Δ+RTA1* constructs. (a) *rta1Δ* construct in the pCR-Blunt II TOPO vector (Invitrogen, Waltham, MA, USA). (b) *rta1Δ+RTA1* construct in the pAllet vector (a kind gift of Dr. James Robertson).

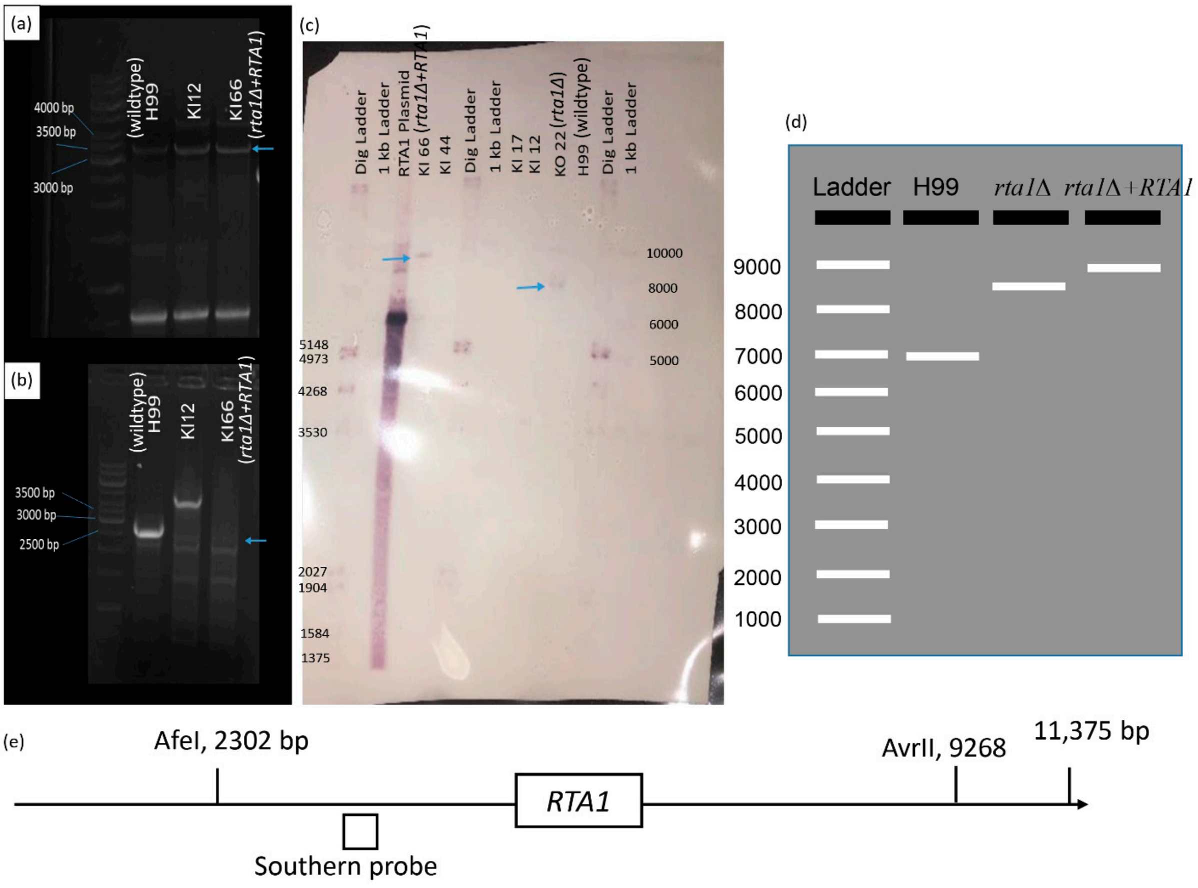

Figure S3. Confirmation of *rta1Δ+RTA1* construct insertion into *C. neoformans* using PCR and Southern blot analysis. For all, H99 is the wildtype control and KI66 is the *rta1Δ+RTA1* strain. a) PCR confirmation that the gene and upstream region were inserted into *C. neoformans*. Anticipated PCR band size is 3633 bp. The blue arrow indicates the correct size band. b) PCR confirmation that half of the gene and the downstream region was inserted into *C. neoformans*. Anticipated band size is 2717 bp. The blue arrow indicates the correct size band. c) Southern blot analysis confirming that *RTA1* is present in the *rta1Δ+RTA1* strain (KI66) created and carried forward into phenotypic testing. Blue arrows indicate the correct size bands in the *rta1Δ+RTA1* and *rta1Δ* strains. (d) Schematic of Southern blot DNA digested with *Afe*I and *Avr*II. H99 is 6966 bp, *rta1Δ* is 8597 bp, and *rta1Δ+RTA1* is 8966 bp. *rta1Δ* contains noursethricin and *rta1Δ+RTA1* contains neomycin. (e) Restriction map of genomic DNA.

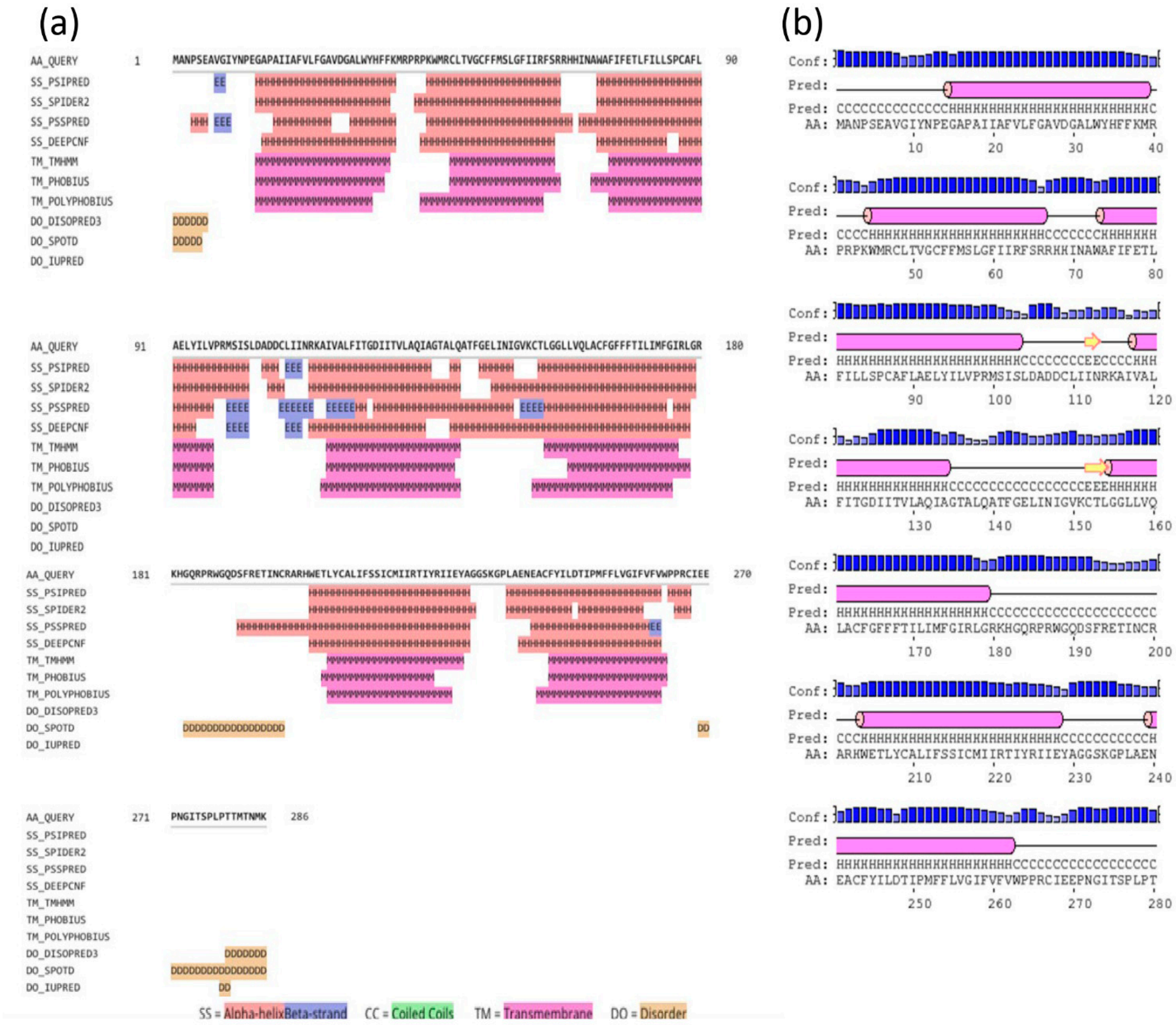

Figure S4. Secondary structure protein modeling for Rta1. To predict Rta1 secondary structure, the amino acid coding sequence was input into protein modeling software. (a) Analysis of putative secondary structure using Quick2D software shows seven theoretical transmembrane regions and predicts that Rta1 is mostly comprised of alpha-helices. Quick2D software incorporates data from several programs to generate multiple secondary structure models. (b) The same analysis was completed using only PsiPred, which is the first entry from the Quick2D software, to demonstrate more clearly where the 7 alpha helices (pink) are predicted to be for Rta1. The blue bars indicate the confidence value at each position.

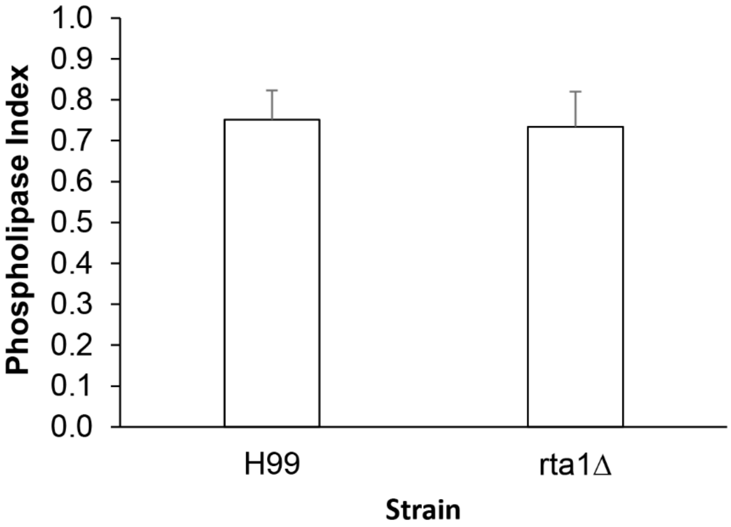

Figure S5. Phospholipase index of H99 and *rta1Δ*. Colonies were grown on YPD media + malt egg yolk agar for 10 days at 30 °C and the diameter of the precipitate for 15 colonies across three experiments was measured.

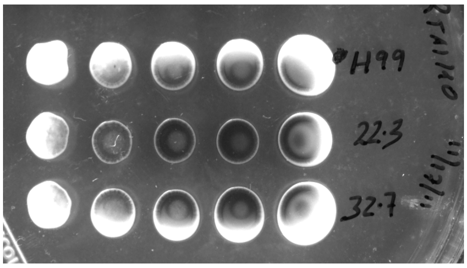

Figure S6. Melanin production of H99 and *rta1Δ*. Dilutions of strains were spotted on minimal medium + L-DOPA and incubated at 30 °C for 3–5 days. N = 3. 22-3 is the *rta1Δ* strain and 32-7 is another *rta1Δ* transformant that was tested.

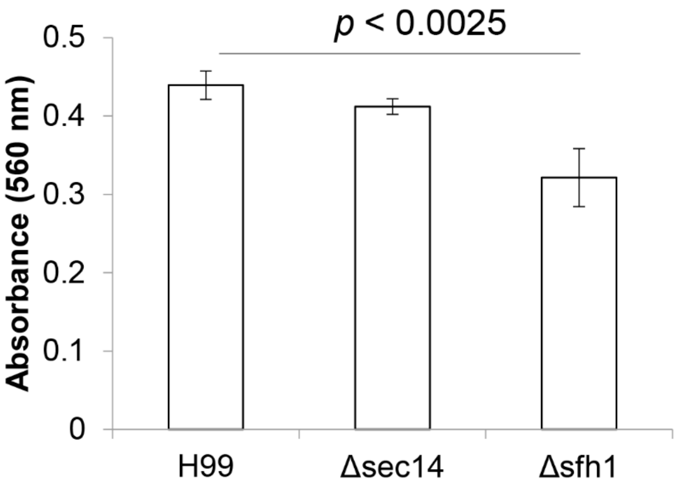

Figure S7. Urease is not secreted via the Sec14 pathway. Information on the origin, construction, and confirmation of deletion of the *Δsec14* and *Δsfh1* strains have been previously published [1]. N = 3.

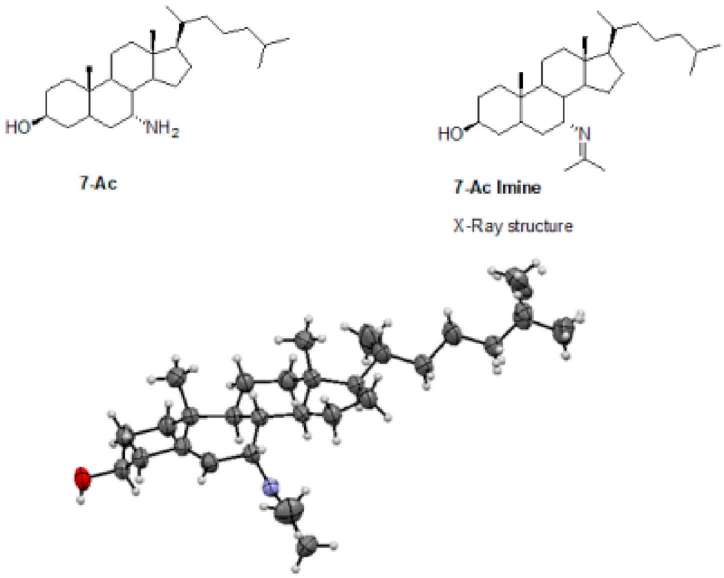

Figure S8. Perspective view of the crystal structure of 7-aminocholesterol generated using Ortep-III [2].

Table S1. List of primers used for amplification and diagnostics of *RTA1*.

Component	Primer sequence	Restriction Enzyme
5' *RTA1* Left 5' UTR + *NAT* 3'	ATATTAGGCCTAATCGATTCAATCTACGCTCTCAC	StuI
3' *RTA1* Left 5' UTR + *NAT* 5'	AAGAGCCAAGGATTCGCGAGTTTCGTGGTTTCAG	
5' *RTA1* Right KO 3'	ctgaaaccacgaaactcgCGAATCCTTGGCTCTTG	
3' *RTA1* Right KO 5'	CAACAAGACAGGTTTTTTGCAGTAATCCCTC	
5' *NAT* Cassette	GGGAAGGAGCGGACTTACAT	
3' *NAT* Cassette	TGTAGAAACGAGTTTCGTGGTTT	
5' Overlap KO PCR primer	ATATTAGGCCTAATCGATTCAATCTACGCTCTCAC	
3' Overlap KO PCR primer	CAACAAGACAGGTTTTTTGCAGTAATCCCTC	
5' *RTA1* 5' UTR	attattGCGGCCGCACCTCTTTCCAGCCAGTCC	NotI
3' *RTA1* 5' UTR	attattGTCGACAAAATGACGTGTTGGCATG	SalI
5' *NEO* Cassette	attattTCTAGAAGCGGGCAGTGAGCG	XbaI
3' *NEO* Cassette	tataagCTTAAGTGGTTGTCGGAGGAGAGG	AflII
5' *RTA1* 3' Downstream	atattaCCTGCAGGGACATACAGAGGATGAGC	SbfI
3' *RTA1* 3' Downstream	gtaataGCTAGCATAATAGTCAGGCCGGCAC	NheI
5' Mid *RTA1*	GTTCTCGCTCAAATTGCTGG	
3' Mid *RTA1*	CCAGCAATTTGAGCGAGAAC	
5' 2 KB Outside *RTA1*	CGTTCCAGTCGGGGATGGTATT	
3' 2 KB Outside *RTA1*	AAGCCACTGAGTATGACGTCTA	
DIG Probe 5' Primer	GGGGGTTGGAGAATTCTTATGGGATG	
DIG Probe 3' Primer	CATTCTATTTTCCCGAGCTACTGAGAC	
5′ CNAG_00483 (*ACT1*) for qPCR	GCCCAGTCTTCTCAGCTTGAAA	
3′ CNAG_00483 (*ACT1*) for qPCR	ACTTTCGGTGGACGATTGAGG	
5′ CNAG_03091 (*RTA1*) for qPCR	GATCAATATCGGTGTGAAGTGTACC	
3′ CNAG_03091 (*RTA1*) for qPCR	TCAACGCACAGTACAACGTCT	
5′ CNAG_01911 (*SEC6*) for qPCR*	GCCAGAAGAACCTACAATTATGGTC	
3′ CNAG_01911 (*SEC6*) for qPCR*	GCGAAGTTGCTAGCATATGTTCC	

**SEC6* primers were designed to avoid the region used in the iSEC6 interference vector.

Table S2. Crystal data, details of data collection and refinement parameters for 7-aminocholesterol.

Crystal Information	
Chemical Formula	$C_{30} H_{57} N O$
Formula Weight	441.72
Crystal System	Monoclinic
Space group	C2
a (Å)	21.7123(5)
b (Å)	7.9327(2)
c (Å)	19.0021(5)
β (°)	121.529(1)
Z	4
Intensity data collection & refinement	
Wavelength (Å)	0.71073
Data collection temperature (°K)	293
Total number of reflections	13,329
Unique reflections	3,602
Unique reflections with I > 2σ	2,642
θ range (°)	2.82 to 28.5
R factor (I > 2σ)	0.0544
wR2 (all data)	0.1442
Goodness of fit on F	1.118

References

1. Chayakulkeeree, M.; Johnston, S.A.; Oei, J.B.; Lev, S.; Williamson, P.R.; Wilson, C.F.; Zuo, X.; Leal, A.L.; Vainstein, M.H.; Meyer, W., et al. SEC14 is a specific requirement for secretion of phospholipase B1 and pathogenicity of Cryptococcus neoformans. *Mol Microbiol* **2011**, *80*, 1088-1101, doi:10.1111/j.1365-2958.2011.07632.x.
2. Farrugia, L.J. ORTEP-3 for Windows – a version of ORTEPIII with a Graphical user Interface (GUI). *J Appl Cryst* **1997**, *30*, 565, doi: https://doi.org/10.1107/S0021889897003117.
